# Supplementary material for: Inhibiting WNT Ligand Production for Improved Immune Recognition in the Ovarian Tumor Microenvironment
Source: Cancers (Basel). 2020 Mar 24;12(3):766. doi: 10.3390/cancers12030766 (PMC7140065; doi:10.3390/cancers12030766)
Supplement: Supplementary file 1 [file cancers-12-00766-s001.pdf]

*Supplementary Materials*

# Inhibiting WNT Ligand Production for Improved Immune Recognition in the Ovarian Tumor Microenvironment

**Whitney N. Goldsberry, Selene Meza-Perez, Angelina I. Londoño, Ashwini A. Katre, Bryan T. Mott, Brandon M. Roane, Nidhi Goel, Jaclyn A. Wall, Sara J. Cooper, Lyse A. Norian, Troy D. Randall, Michael J. Birrer and Rebecca C. Arend**

| Gene | Cell Type |
|---|---|
| BLK | B cell |
| CD19 | B cell |
| FCRL2 | B cell |
| MS4A1 | B cell |
| KIAA0125 | B cell |
| TNFRSF17 | B cell |
| TCL1A | B cell |
| SPIB | B cell |
| PNOC | B cell |
| PTPRC | $CD45^+$ |
| PRF1 | Cytotoxic Cells |
| GZMA | Cytotoxic Cells |
| GZMB | Cytotoxic Cells |
| NKG7 | Cytotoxic Cells |
| GZMH | Cytotoxic Cells |
| KLRK1 | Cytotoxic Cells |
| KLRB1 | Cytotoxic Cells |
| KLRD1 | Cytotoxic Cells |
| CTSW | Cytotoxic Cells |
| GNLY | Cytotoxic Cells |
| LAG3 | Exhausted $CD8^+$ |
| CD244 | Exhausted $CD8^+$ |
| EOMES | Exhausted $CD8^+$ |
| PTGER4 | Exhausted $CD8^+$ |
| CD68 | Macrophage |
| CD84 | Macrophage |
| CD163 | Macrophage |
| MS4A4A | Macrophage |
| FPR1 | Neutrophil |
| SIGLEC5 | Neutrophil |
| CSF3R | Neutrophil |

| | |
|---|---|
| FCAR | Neutrophil |
| FCGR3B | Neutrophil |
| CEACAM3 | Neutrophil |
| S100A12 | Neutrophil |
| KIR2DL3 | NK CD56dim |
| KIR3DL1 | NK CD56dim |
| KIR3DL2 | NK CD56dim |
| IL21R | NK CD56dim |
| XCL1 | NK cells |
| XCL2 | NK cells |
| NCR1 | NK cells |
| CD6 | T cell |
| CD3D | T cell |
| CD3E | T cell |
| SH2D1A | T cell |
| TRAT1 | T cell |
| CD3G | T cell |
| TBX21 | Th1 |
| CD8A | $CD8^+$ T cell |

(a)

| **WNT Gene** |
|---|
| TH-isoform1 |
| MSX1 |
| WNT11 |
| FGF9 |
| CLDN2 |
| GLI2 |
| TMSB15A |
| COL4A5 |
| COL4A6 |
| NCAM1 |
| SOX5 |
| MYOD1 |
| FOXA2 |
| IHH |
| GLI1 |
| TSPAN8 |
| CYP11A1 |
| TDGF1 |

| |
|---|
| LHCGR |
| CTNND2 |
| WIF1 |
| EDN3 |
| PCSK6-isoform1 |
| PCSK6-isoform2 |
| GFAP |
| CST4 |
| CDH2 |
| SOX11 |
| CTNNA2 |
| EMX2 |
| BMP7 |
| LGR5 |
| MUC6 |
| SHH |
| SIM2 |
| TH-isoform2 |

(b)

| **RIN Values** |
|---|
| 8.4 |
| 7.8 |
| 6.0 |
| 8.5 |
| 8.9 |
| 8.1 |
| 7.5 |
| 7.2 |
| 8.3 |
| 5.8 |
| 2.4 |
| 8.4 |
| 8.1 |
| 8.4 |
| 8.7 |
| 3.1 |
| 8.3 |
| 6.2 |

(c)

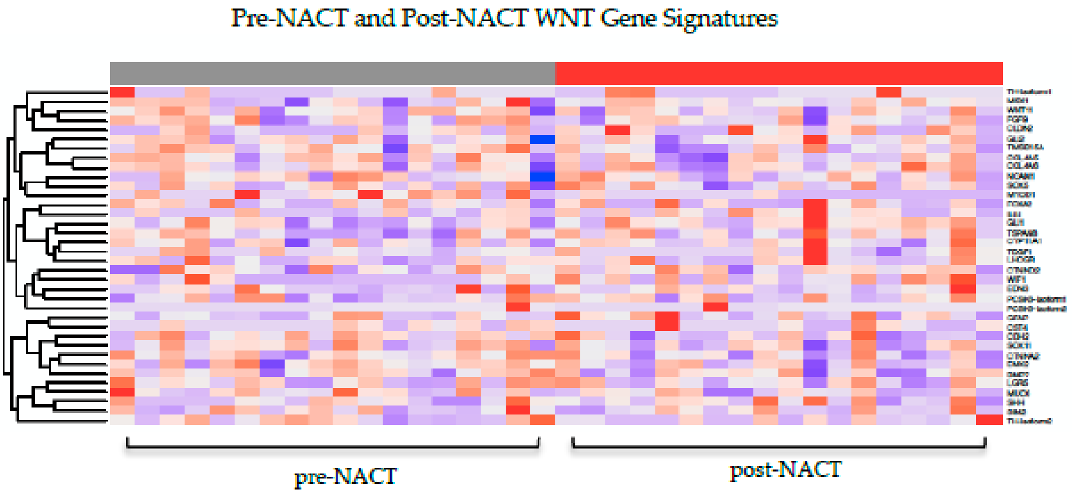


(d)

| Pre-NACT | Post-NACT | WNT fold change | PFS (months) |
|---|---|---|---|
| Hot | Hot | 0.88 | 33.2 |
| Cold | Intermediate | 0.85 | 25.7 |
| Hot | Hot | 0.96 | 22.7 |
| Intermediate | Hot | 1.03 | 19.7 |
| Hot | Intermediate | 0.95 | 17.6 |
| Cold | Hot | 0.99 | 12.6 |
| Intermediate | Hot | 1.06 | 10.0 |
| Intermediate | Hot | 1.04 | 9.5 |
| Intermediate | Cold | 1.19 | 9.2 |
| Cold | Cold | 1.05 | 8.2 |
| Cold | Hot | 0.92 | 6.9 |
| Intermediate | Cold | 1.04 | 6.8 |
| Cold | Cold | 1.01 | 6.4 |
| Hot | Hot | 1.00 | 5.1 |
| Intermediate | Intermediate | 1.05 | 4.2 |

| | | | |
|---|---|---|---|
| Cold | Cold | 1.00 | 4.0 |
| Hot | Cold | 0.97 | 3.7 |

(e)

**Figure S1.** Wnt and immune gene signature in ovarian cancer patient samples. (**a**) Genes used to determine T cell signature with associated cell type. (**b**) Genes used to determine WNT activity. (**c**) RNA Integrity Numbers (RIN) for samples that were measured. (**d**) Heatmap with matched pre-NACT and post-NACT WNT gene signatures. (**e**) Matched pre-NACT and post-NACT T cell signature assignments, WNT fold change, and PFS.

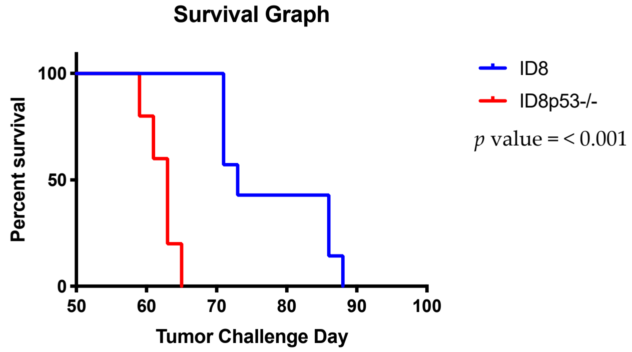


(a)

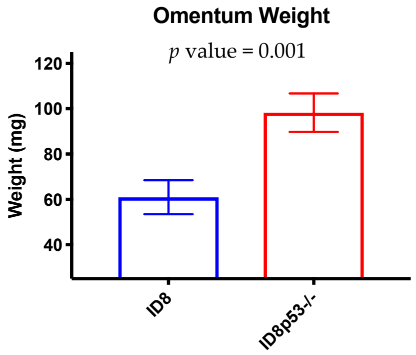


(b)

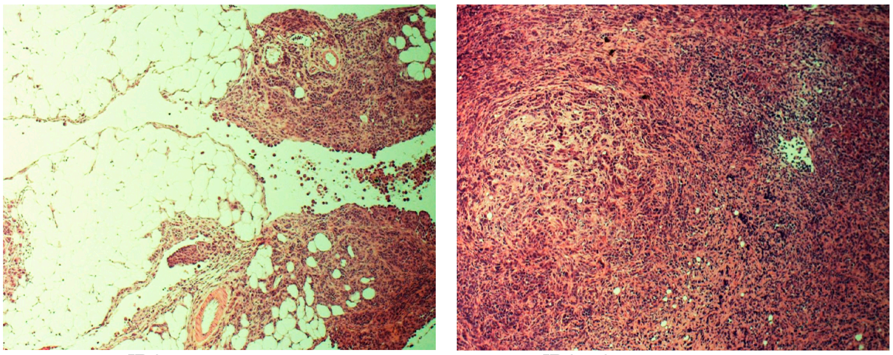

ID8 omentum tumor ID8p53$^{-/-}$ omentum tumor

(c)

**Figure S2.** ID8 and ID8p53$^{-/-}$ differences of survival and tumor burden. (**a**) Survival was increased with ID8 intraperitoneal tumor challenge in C57Bl/6 mice compared with ID8p53$^{-/-}$ tumor challenge. (**b**) On tumor challenge day 42, omentum weights between ID8 and ID8p53$^{-/-}$ tumor challenge were statistically different. (**c**) Hematoxylin and eosin stained formalin fixed paraffin embedded tissue samples of omentum tumor after 42 days of tumor challenge with ID8 or ID8p53$^{-/-}$ cells.

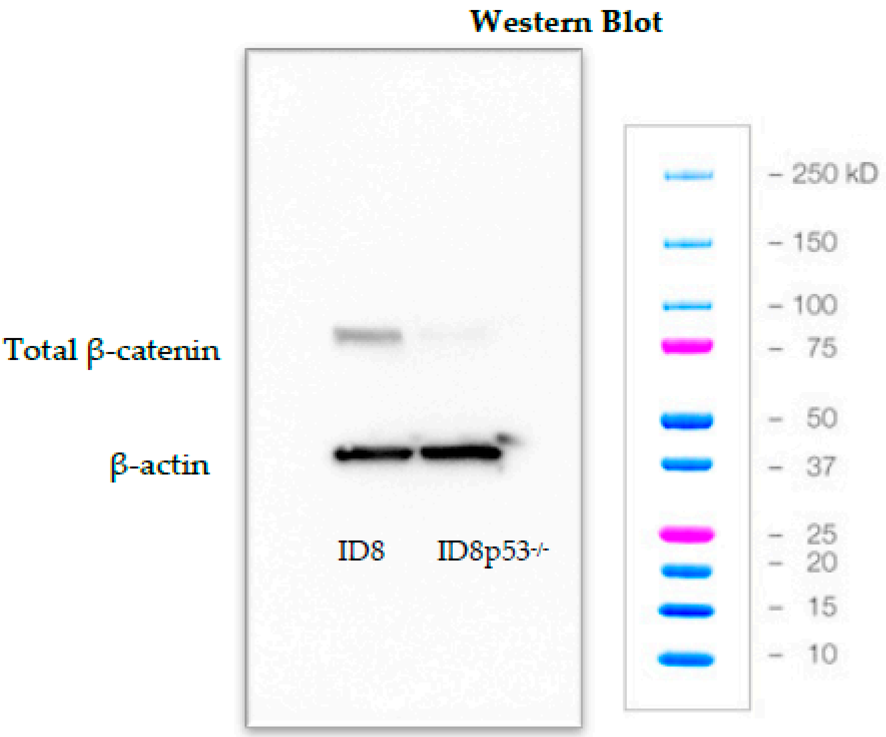


(a)

**Figure S3.** Comprehensive Western blot. (**a**) Reduced baseline β–catenin levels in the ID8p53-/- cell line are evident with control β–actin levels and molecular weight ladder.

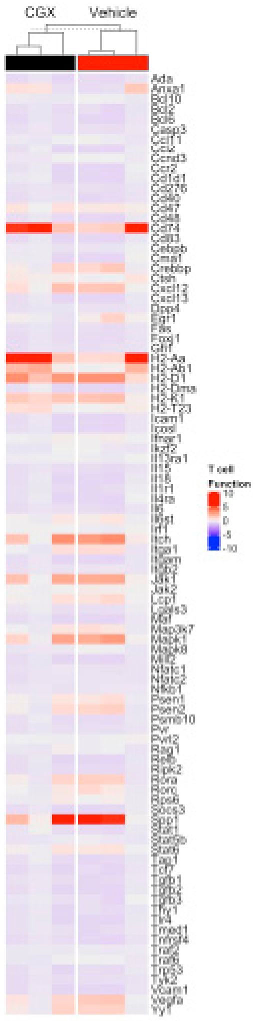
CGX
Vehicle
Ada
Anxa1
Bcl10
Casp3
Ccnd3
Ccr2
Cd1d1
Cd276
Cd74
Cebpb
Crebbp
Cxcl12
Cxcl13
Dpp4
Egr1
Foxp1
H2-Aa
H2-Ab1
H2-D1
H2-Dma
H2-K1
H2-T23
Icam1
Icosl
Ifnar1
Ikzf2
Il13ra1
Il1r1
Il4ra
Itch
Itga1
Jak1
Jak2
Lcp1
Lgals3
Map3k7
Mapk1
Mapk8
Nfatc1
Nfatc2
Nfkb1
Psen1
Psen2
Psmb10
Pvr
Pvrl2
Rag1
Relb
Ripk2
Rora
Rorc
Socs3
Spp1
Tgfb1
Thy1
Tmed1
Tnfrsf4
Tp53
Tyk2
Vcam1
Vegfa
Yy1
T cell
Function
10
5
0
-5
-10

(a)

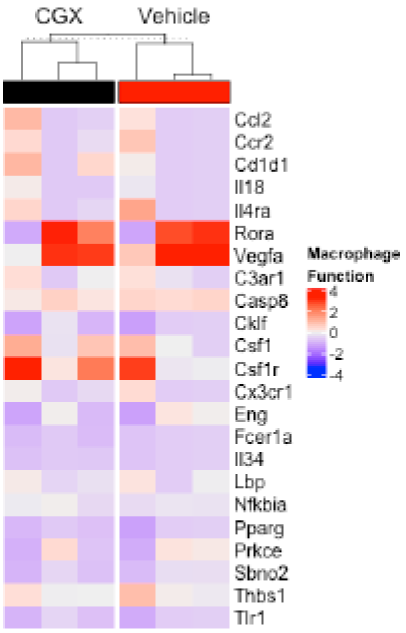
CGX
Vehicle
Ccl2
Ccr2
Cd1d1
Il18
Il4ra
Rora
Vegfa
C3ar1
Casp8
Cklf
Csf1
Csf1r
Cx3cr1
Eng
Fcer1a
Il34
Lbp
Nfkbia
Pparg
Prkce
Sbno2
Thbs1
Tlr1
Macrophage Function
4
2
0
-2
-4

(b)

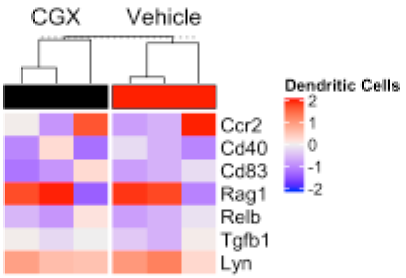
CGX
Vehicle
Ccr2
Cd40
Cd83
Rag1
Relb
Tgfb1
Lyn
Dendritic Cells
2
1
0
-1
-2

(c)

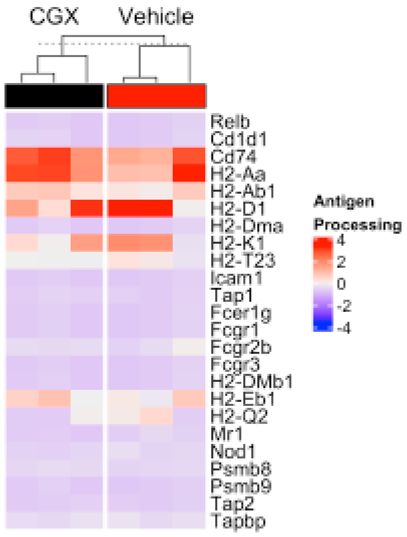
CGX
Vehicle
Relb
Cd1d1
Cd74
H2-Aa
H2-Ab1
H2-D1
H2-Dma
H2-K1
H2-T23
Icam1
Tap1
Fcer1g
Fcgr1
Fcgr2b
Fcgr3
H2-DMb1
H2-Eb1
H2-Q2
Mr1
Nod1
Psmb8
Psmb9
Tap2
Tapbp
Antigen Processing
4
2
0
-2
-4

(d)

**Figure S4.** *In vivo* effect of Wnt signaling inhibition in omentum tumor and the microenvironment. Heatmaps, with signature gene lists, associated with each pathway signature score for NanoString analysis of omentum at 42 days tumor challenge with ID8 cells, with or without CGX1321 treatment, including gene signatures for T cell functions (**a**), macrophage functions (**b**), dendritic cell functions (**c**), and antigen processing (**d**).

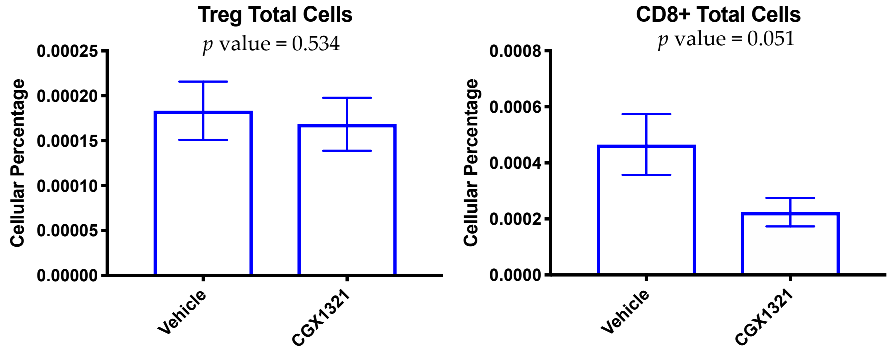


(a)

**Figure S5.** *In vivo* effect of Wnt signaling inhibition in omentum tumor and the microenvironment. (**a**) After 42 days of ID8 tumor challenge in C56Bl/6 mice, flow cytometry of omentum tumor did not reveal a statistical difference in Treg and CD8+ T cellular percentages with CGX1321 treatment.

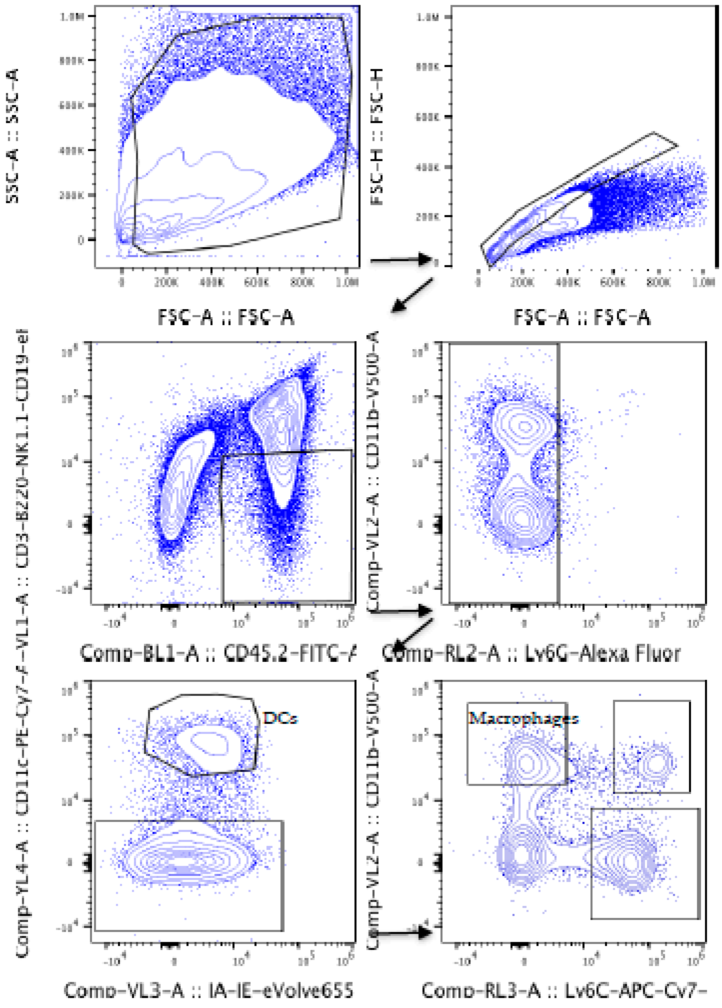


(a)

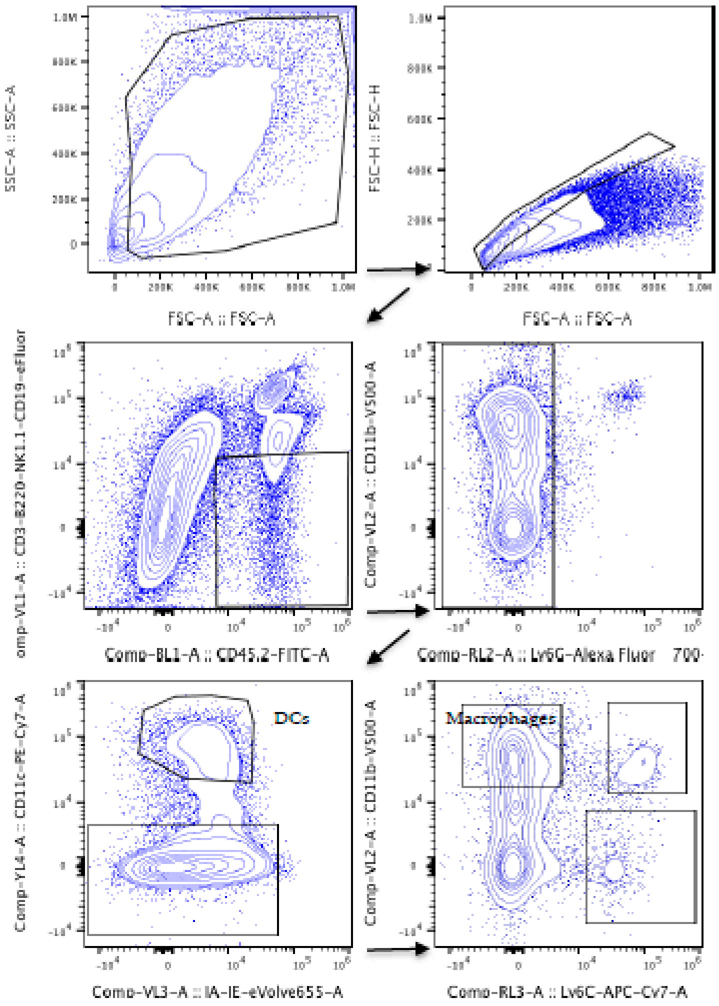
SSC-A :: SSC-A
FSC-A :: FSC-A
FSC-H :: FSC-H
FSC-A :: FSC-A
omp-VL1-A :: CD3-B220-NK1.1-CD19-eFluor
Comp-BL1-A :: CD45.2-FITC-A
Comp-VL2-A :: CD11b-V500-A
Comp-RL2-A :: Ly6G-Alexa Fluor 700-
Comp-YL4-A :: CD11c-PE-Cy7-A
DCs
Comp-VL3-A :: IA-IE-eVolve655-A
Comp-VL2-A :: CD11b-V500-A
Macrophages
Comp-RL3-A :: Ly6C-APC-Cy7-A

(c)

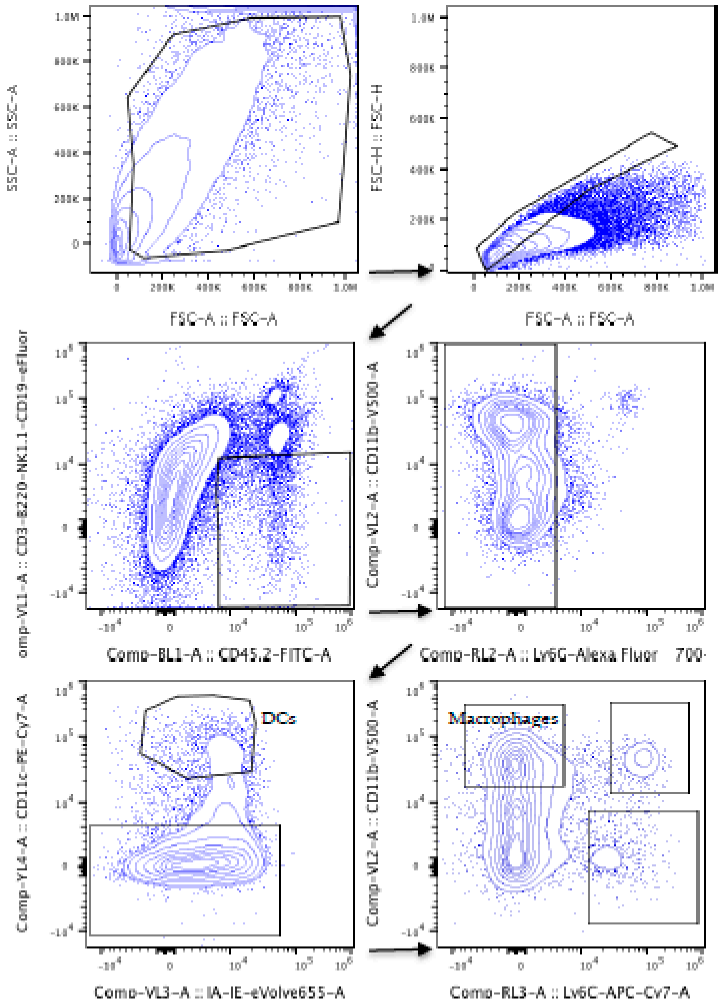


(b)

**Figure S6.** DC and macrophage flow cytometry gating strategy. Cell population selection narrowed from left to right, as displayed by black arrows. (**a**) Gating strategy for naive mouse omentum, without tumor. (**b**) Gating strategy for omentum tumor with mice treated with vehicle. (**c**) Gating strategy for omentum tumor for mice treated with CGX1321.

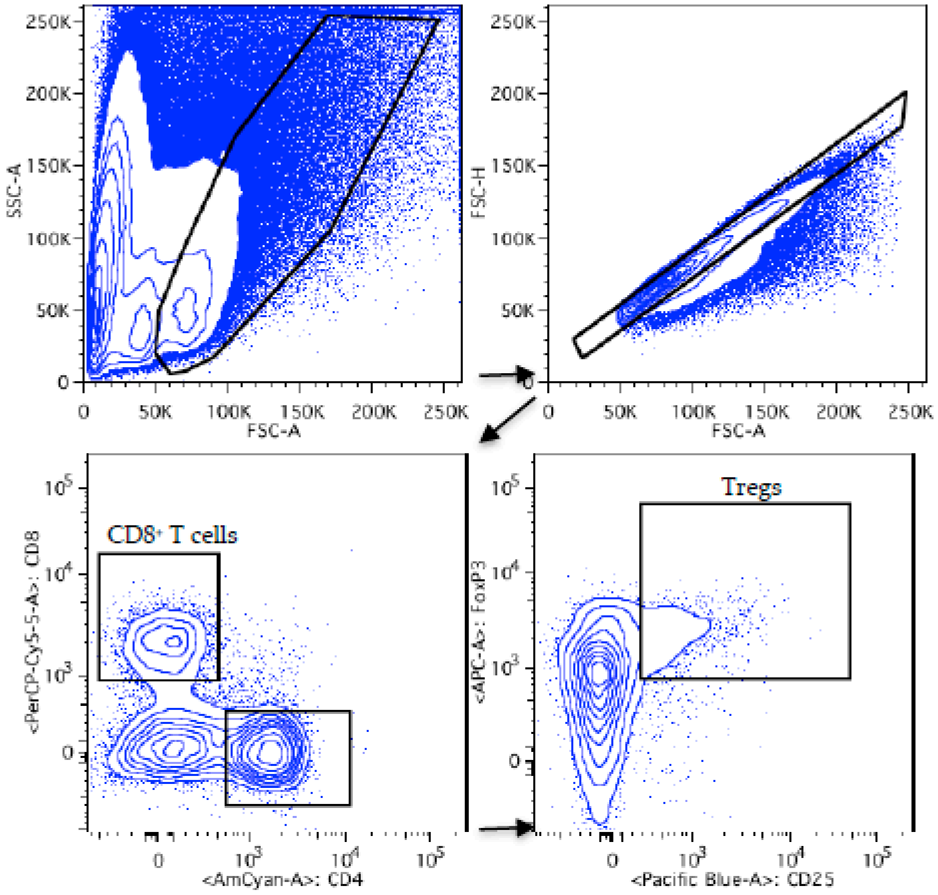

SSC-A
FSC-A
FSC-H
FSC-A
CD8⁺ T cells
<PerCP-Cy5-5-A>: CD8
<AmCyan-A>: CD4
Tregs
<APC-A>: FoxP3
<Pacific Blue-A>: CD25


(a)

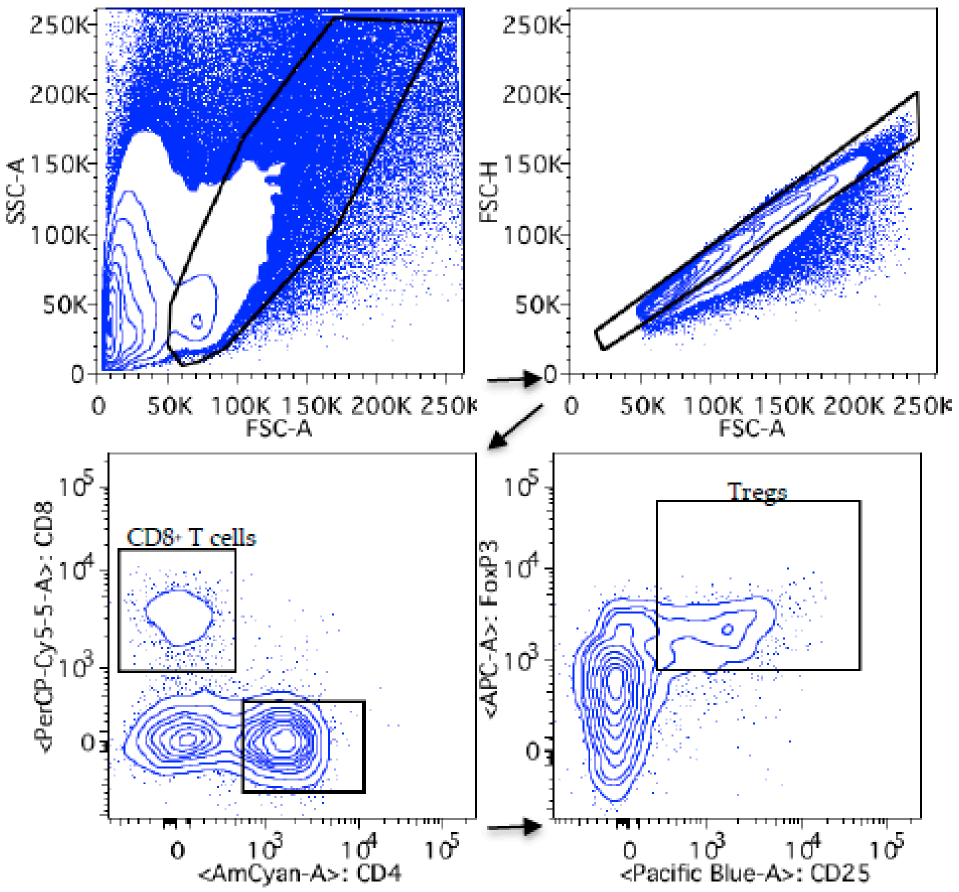

250K
200K
150K
100K
50K
0
SSC-A
0
50K
100K
150K
200K
250K
FSC-A
FSC-H
CD8+ T cells
<PerCP-Cy5-5-A>: CD8
<AmCyan-A>: CD4
Tregs
<APC-A>: FoxP3
<Pacific Blue-A>: CD25


(b)

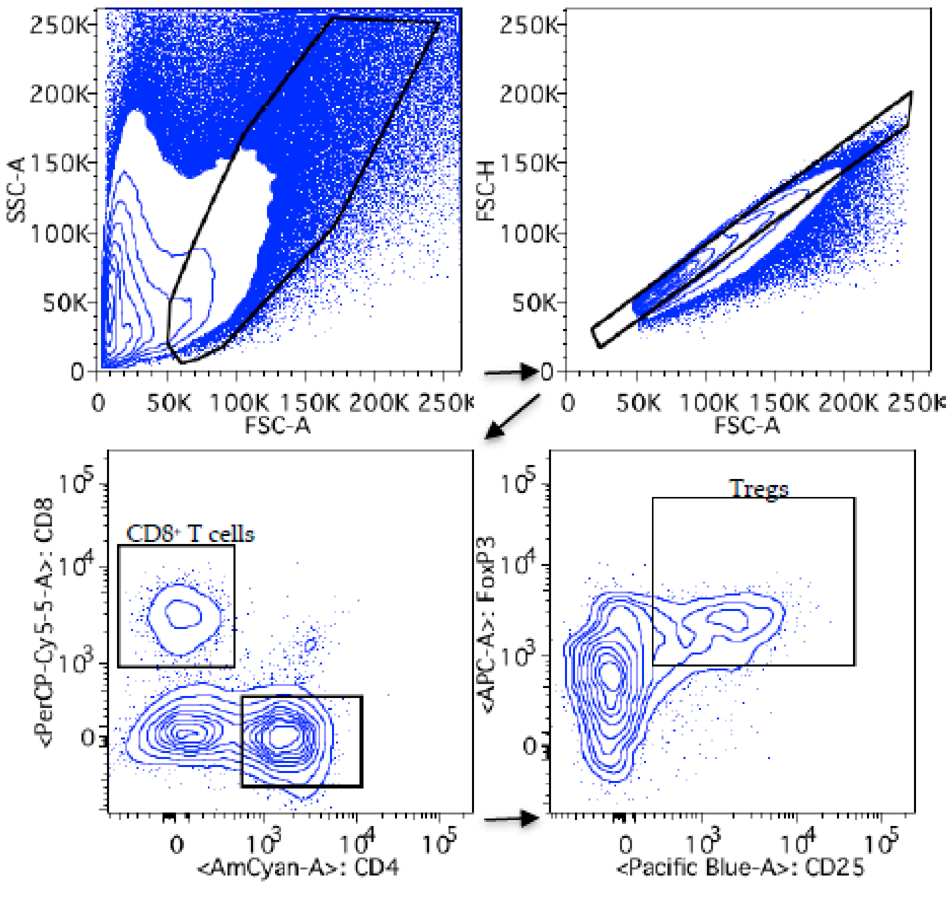


(c)

**Figure S7.** Treg and CD8+ T cell flow cytometry gating strategy. Cell population selection narrowed from left to right, as displayed by black arrows. (**a**) Gating strategy for naive mouse omentum, without tumor. (**b**) Gating strategy for β–catenin$^{-/fl/fl}$ mouse omentum tumor. (**c**) Gating strategy for CD11c-cre x β– catenin$^{-/fl/fl}$ mouse omentum tumor.

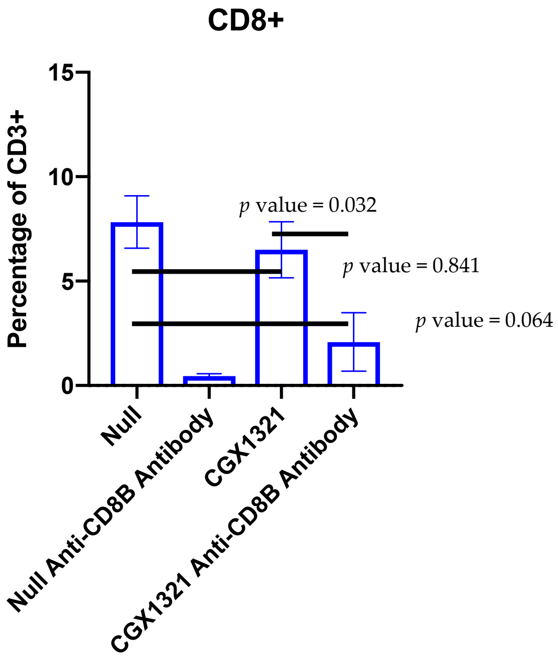

(a)

**Figure S8.** Anti-CD8+β antibody with decreased CD8+ T cells. (**a**) Flow cytometry confirmed a decrease in CD8+ T cells in the omentum of mice treated with anti-CD8+β antibody.

© 2020 by the authors. Licensee MDPI, Basel, Switzerland. This article is an open access article distributed under the terms and conditions of the Creative Commons Attribution (CC BY) license (http://creativecommons.org/licenses/by/4.0/).
